# Growth hormone/IGF-I-dependent signaling restores decreased expression of the myokine SPARC in aged skeletal muscle

**DOI:** 10.1007/s00109-022-02260-w

**Published:** 2022-09-30

**Authors:** Sebastian Mathes, Alexandra Fahrner, Edlira Luca, Jan Krützfeldt

**Affiliations:** 1 Department of Endocrinology, Diabetology, and Clinical Nutrition, University Hospital Zurich (USZ), University of Zurich (UZH), Rämistrasse 100, 8091 Zurich, Switzerland; 2 Life Science Zurich Graduate School, Biomedicine, University of Zurich, 8057 Zurich, Switzerland

**Keywords:** Skeletal muscle, IGF-I, SPARC, IMAT, Aging, Growth hormone

## Abstract

**Abstract:**

Skeletal muscle exerts many beneficial effects on the human body including the contraction-dependent secretion of peptides termed myokines. We have recently connected the myokine secreted protein acidic and rich in cysteine (SPARC) to the formation of intramuscular adipose tissue (IMAT) in skeletal muscle from aged mice and humans. Here, we searched for inducers of SPARC in order to uncover novel treatment approaches for IMAT. Endurance exercise in mice as well as forskolin treatment in vitro only modestly activated SPARC levels. However, through pharmacological treatments in vitro, we identified IGF-I as a potent inducer of SPARC expression in muscle cells, likely through a direct activation of its promoter via phosphatidylinositol 4,5-bisphospate 3-kinase (PI3K)-dependent signaling. We employed two different mouse models of growth hormone (GH)/IGF-I deficiency to solidify our understanding of the relationship between IGF-I and SPARC in vivo. GH administration robustly increased intramuscular SPARC levels (3.5-fold) in GH releasing hormone receptor-deficient mice and restored low intramuscular SPARC expression in skeletal muscle from aged mice. Intramuscular glycerol injections induced higher levels of adipocyte markers (adiponectin, perilipin) in aged compared to young mice, which was not prevented by GH treatment. Our study provides a roadmap for the study of myokine regulation during aging and demonstrates that the GH/IGF-I axis is critical for SPARC expression in skeletal muscle. Although GH treatment did not prevent IMAT formation in the glycerol model, targeting SPARC by exercise or by activation of IGF-I signaling might offer a novel therapeutic strategy against IMAT formation during aging.

****Key messages**:**

- IGF-I regulates the myokine SPARC in muscle cells directly at the promoter level.
- GH/IGF-I is able to restore the decreased SPARC levels in aged skeletal muscle.
- The glycerol model induces higher adipocyte markers in aged compared to young muscle.
- GH treatment does not prevent IMAT formation in the glycerol model.

**Supplementary Information:**

The online version contains supplementary material available at 10.1007/s00109-022-02260-w.

## Introduction

Skeletal muscle is an important tissue for posture, movement, energy expenditure, and glucose disposal in the human body. The contraction-dependent secretion of peptides termed myokines is thought to be an important part of the health benefits of muscle tissue and the adaptive responses to exercise [1]. Myokines have the potential to act as autocrine, paracrine, or endocrine signaling mediators in numerous metabolic processes, as well as in muscle regeneration and inflammation [2]. During the aging process muscle mass and function continuously decline, which has detrimental consequences for human health [3]. However, the regulation of myokines during the aging process remains poorly understood. For example, the negative regulator of myogenesis myostatin was reported in aged humans to be increased [4] or unchanged [5], while the expression of follistatin, an antagonist of myostatin, was unchanged during the aging process [6].

The aging process leads to a redistribution of adipose tissue depots resulting in changes in adipose storage locations from subcutaneous to more harmful ectopic sites such as skeletal muscle [7, 8]. The intramuscular adipose tissue (IMAT) positively correlates with aging [9–12] and is associated with insulin resistance [13, 14], decreased muscle strength [15], and mobility dysfunction [16–18]. The amount of IMAT can be substantial and is estimated to relate in obese subjects to 5% (women) or 10% (men) of whole-body adipose tissue [13]. The molecular cues that regulate IMAT are poorly understood. We have previously shown that activated fibroblast growth factor-2 (FGF-2) signaling induces IMAT by suppressing the myokine SPARC in aged skeletal muscle via microRNA (miR) 29a [19]. SPARC (also known as osteonectin or BM-40) is a 43 kDa calcium binding, matricellular glycoprotein that is secreted by several cell types and is involved in extracellular matrix reorganization, cell adhesion, and proliferation [20]. SPARC has been demonstrated to act as a myokine in both mice [21] and humans [22, 23]. Multiple biological processes are affected by SPARC besides IMAT formation; for example, it participates in wound healing [24], tissue response to injury [25], angiogenesis [26], tumorigenesis [27], and inflammation [28].

The molecular cues that govern the expression of this important myokine besides miR-29a, especially in skeletal muscle during aging, still remain largely unknown. Here, we show that insulin-like growth factor I (IGF-I) activates the human SPARC promoter and consequently increases SPARC expression in muscle cells. Systemic stimulation of IGF-I levels via growth hormone replacement therapy (GHRT) markedly increased SPARC levels in the skeletal muscle of a mouse model for GH deficiency, while GH administration restored decreased SPARC levels in aged skeletal muscle. Together, we propose IGF-I as potent modulator of SPARC that might counteract FGF-2/miR-29a signaling to regulate ectopic fat formation in the aged skeletal muscle. Thus, our data provide novel insights that could be implemented in future strategies to prevent IMAT formation during aging.

## Material and methods

### Animals

All animals used were male mice housed at 3–5 littermates per cage in individually ventilated cages under conditions of controlled temperature (22 °C) and illumination (12-h light/12-h dark cycle; light off at 6 p.m.) with ad libitum access to chow and water. Health status of all mouse lines was monitored on a regular basis according to FELASA guidelines. All animal procedures were approved by the Veterinary Office of the Canton of Zurich. Wildtype C57BL/6 mice (Envigo) were exercised on a five-lane mouse treadmill (Panlab, Harvard Bioscience) with a gradient angle of 14° for 90 min, comprising a warm-up phase starting at 10 cm/s followed by a gradual increment in speed (1 cm/s every 30 s) until the target speed of 23 cm/s was reached and maintained until the end of the session. Young control (2 months) and aged (25 months) C57BL/6JRj (Janvier) or C57BL/6 J-growth hormone releasing hormone receptor (Ghrhr)^lit^/J mice (Jackson) between 10 and 14 weeks of age were injected s c. once daily with recombinant growth hormone (GH; Genotropin, Pfizer) at a dose of 6 µg/g bodyweight per day for 21 days.

### Cell culture

C2C12 cells were cultured in DMEM supplemented with 10% FBS (Gibco) and 1% penicillin/streptomycin (P/S, Gibco). Differentiation was initiated when myoblasts reached 90% confluency by changing the media to DMEM containing 2% horse serum (Gibco) and 1% P/S. After 4 days of differentiation under low-serum conditions, fully differentiated myotubes were treated for 6 h with 1–100 µM forskolin, NKH477 (Calbiochem), or for 48 h with 50 µM dexamethasone (Sigma), 100 nM IGF-I, 2.7 nM Wnt-3a, 578 pM TNF-α, 20 nM GDF-8, or 640 pM IFN-γ (all purchased from PeproTech) in starvation medium containing 0.5% FBS. For inhibitor assays, C2C12 myoblasts or myotubes were treated with 10 µM PD184352, 50 µM NSC23766, 20 µM LY294002, 10 µM U73122, and 12.5 µM VX-509 for 48 h, as indicated. All inhibitors were purchased from Selleckchem except for NSC23766 (Sigma). For miR29a inhibition assay, C2C12 myotubes were transfected (Lipofectamine RNAiMAX; Invitrogen) with 12 nM antagomirs (Sigma). When indicated, cells were treated with 100 nM murine IGF-I or 1 ng/µl growth hormone (Genotropin, Pfizer) for 48 h.

### Plasmid construction, RNAi, and luciferase assay

Human SPARC promoter sequence was PCR-amplified using Phusion High-Fidelity DNA Polymerase (Invitrogen) from human gDNA isolated from primary human myoblasts and cloned into pCR2.1-TOPO using TOPO TA Cloning Kit (Invitrogen). Subsequently, SPARC promoter sequence was ligated into the KpnI/XhoI restriction sites of pGL4.25 [*luc2CP*/minP] (Promega). For promoter assays, C2C12 myoblasts were co-transfected (Lipofectamine 2000; Invitrogen) with pGL4.25-SPARC-Promoter [− 977/ + 35] and pRL-TK (Promega), which provides constitutive expression of *Renilla* luciferase for internal control. For RNAi, myoblasts were triple-transfected with pGL4.25-SPARC-Promoter, pRL-TK. and 200 ng/µl esiRNA (eGFP #EHUEGFP, Akt2 #EMU047871; Sigma). Twenty-four hours after transfection, cells were treated for 48 h with 0.01–1 µM IGF-I or with 100 nM IGF-I and respective inhibitors when indicated as described before. Luciferase reporter activity was measured using the Dual-Luciferase Reporter Assay System (Promega) on an Infinite 200 PRO multimode plate reader equipped with i-control software (Tecan). *Firefly* luciferase values were normalized to *Renilla* luciferase counts. Primers used for plasmid construction are listed in Table S1.

### ELISA

Tail-vein blood was immediately collected after exercise. Heart blood of Ghrhr^lit^/J, young, and aged mice was immediately collected after euthanasia. Cell culture supernatant was collected from C2C12 myotubes 24 h after treatment with 10 µM forskolin. All samples were immediately centrifuged for 10 min at 4 °C. SPARC and IGF-I levels were determined using ELISA Kits for osteonectin (#SEA791Mu, Cloud-Clone Corp.) or IGF-I (#RAB0229, Sigma). Colorimetric solutions were quantified using a PowerWave 340 Microplate Spectrophotometer (BioTek) and Gen5 software v1.11.

### RNA extraction, cDNA synthesis, and quantitative RT-PCR

For RNA isolation from tibialis anterior and gastrocnemius muscle, tissue was dissected immediately after euthanasia of the animals, snap frozen in liquid N2, and stored at − 80 °C until processing. Total RNA from both tissue and cells was isolated using TRIzol Reagent (Invitrogen) according to the manufacturer’s instruction. Traces of genomic DNA were removed using the DNA-free DNA Removal Kit (Invitrogen). Equal amounts of RNA were reverse-transcribed with random hexamer primers using SuperScript III First-Strand Synthesis System (Invitrogen). Quantitative RT-PCR for mRNA levels was performed on a 7500 FAST Real-time PCR system (Applied Biosystems) using FastStart Universal SYBR Green Master (Roche). Gene expression was calculated using the relative standard curve method and 18S rRNA for normalization. All primer sequences are given in Table S1.

### Histology

Mouse tibialis anterior muscles were fixed in 4% formalin, and embedded in paraffin using the Excelsior AS Tissue Processor (Thermo Scientific). Sections of 4-µm thickness were prepared using a fully automated rotary microtome (RM2255, Leica Biosystems). Muscle tissue sections were deparaffinized, subjected to hematoxylin and eosin (H&E) staining, and mounted in Pertex (Histolab). Sections were scanned using the Axio Scan.Z1 slide scanner (Zeiss) equipped with ZEN Imaging software v3.1. For each mouse, two representative transverse sections from two regions of the mid-belly of the muscles, each separated by 500 µm, were subjected to image and statistical analysis. Intramuscular adipocytes were manually counted using the cell counting tool on ImageJ (v1.52q). Determination of total IMAT per section was performed by selecting the adipocytes using the Wand (tracing) tool and subsequent quantification of the area.

### Statistical analysis

All values are reported as mean ± SEM. Sample sizes were determined on the basis of previous experiments and publications using similar methodologies as well as on observed effect sizes. For in vivo studies, littermates were randomly assigned to treatment groups whenever possible or were age-matched. Cell culture experiments were independently reproduced 2–3 times using different passage numbers. Statistical significance (*p ≤ 0.05; **p ≤ 0.01; ***p ≤ 0.001) was evaluated as specified in the figure legends using GraphPad Prism v8.4.2. Artwork was created in GraphPad Prism, Adobe Illustrator, and Inkscape.

## Results

**Fig. 1:**
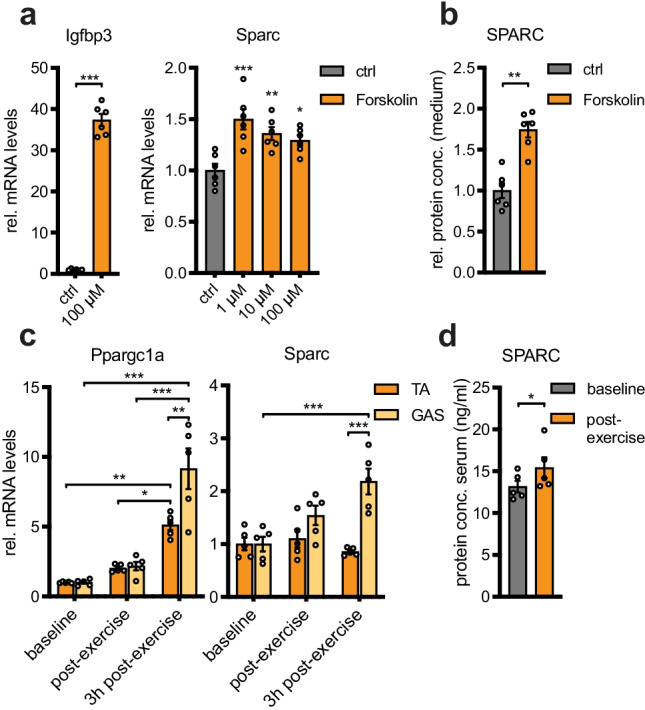
Exercise is a modest regulator of SPARC. **a** Gene expression in C2C12 myotubes after treatment with forskolin for 6 h (*n* = 6). **b** Relative SPARC levels in cell culture medium of C2C12 myotubes after treatment with forskolin for 24 h (*n* = 6). **c** Gene expression in tibialis anterior (TA) and gastrocnemius (GAS) muscle of wildtype C57BL/6 mice before, immediately after, and 3 h after 90 min of running exercise (*n* = 5). **d** Serum SPARC concentration in wildtype C57BL/6 mice before and immediately after 90 min of running exercise as assessed by ELISA (*n* = 5). qPCR values in **a** and **c** were normalized to 18S rRNA. All data are plotted as mean ± SEM. Significance was evaluated by **a** (Igfbp3), **b**, **d** two-tailed unpaired Student’s *t* test, **a** (SPARC) one-way ANOVA with Dunnett’s multiple comparisons test, and **c** two-way ANOVA with Tukey’s multiple comparisons test. *p ≤ 0.05; **p ≤ 0.01; ***p ≤ 0.001

We have previously shown that FGF-2-dependent signaling in aged human skeletal muscle decreases intramuscular SPARC levels via upregulation of miR-29a [19]. Here, our aim was to identify the upstream signaling molecules in skeletal muscle that activate SPARC levels and that could restore SPARC in aged skeletal muscle. First, we tested the effect of exercise as a known stimulus of SPARC expression [21, 23]. To this end, we used an exercise mimetic for studying SPARC expression in vitro and running exercise in vivo. In vitro, we incubated C2C12 myotubes with a derivate (NKH477) of the exercise mimetic forskolin, which is widely used to increase cAMP levels by stimulation of adenylate cyclase activity [29]. Six hours of forskolin treatment stimulated expression of a positive control, insulin-like growth factor-binding protein 3 (Igfbp3), a known exercise responsive gene, as well as induced the expression of SPARC transcript, although to a much lesser degree (Fig. 1a). Forskolin also induced the amount of secreted SPARC in the cell culture medium (Fig. 1b). In vivo, we tested the effect of 90-min running exercise in mice on gene expression in two different skeletal muscle groups, the tibialis anterior (TA) and the gastrocnemius (GAS) muscles. Exercise markedly induced peroxisome proliferator-activated receptor gamma coactivator 1-alpha (Ppargc1a) expression in both muscle groups, while SPARC mRNA levels were only increased in the gastrocnemius muscles (Fig. 1c). In addition, we noted a small but significant increase of circulating SPARC levels immediately after exercise (Fig. 1d). Overall, we conclude that in our protocols, exercise is a modest regulator of SPARC.

**Fig. 2:**
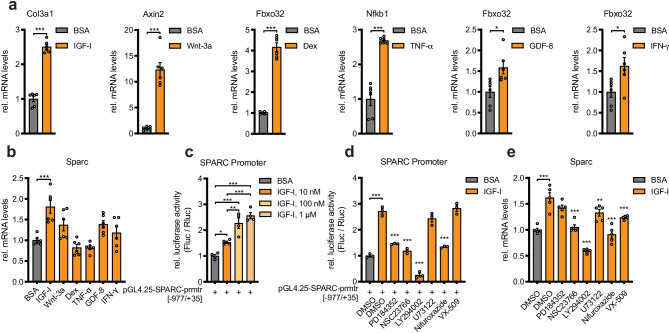
IGF-I increases SPARC expression and activates the SPARC promoter. **a** Gene expression for positive control targets in C2C12 myotubes after treatment with respective recombinant proteins or dexamethasone for 48 h (*n* = 6). **b** SPARC expression from experiment shown in **a** (*n* = 6). **c** Dose curve for human SPARC promoter activity in C2C12 myoblasts after treatment with recombinant IGF-I for 48 h (*n* = 4). **d** Human SPARC promoter activity after treatment with recombinant IGF-I and inhibitors against MEK1/2 (PD184352), Rac GTPase (NSC23766), PI3Kα/δ/β (LY294002), PLC (U73122), STAT1/3/5 (nifuroxazide), or JAK (VX-509). Stars indicate significant difference compared to DMSO control treated with IGF-I (*n* = 3). **e** SPARC expression in C2C12 myotubes after treatment with recombinant IGF-I and respective inhibitors. Stars indicate significant difference compared to DMSO control treated with IGF-I (*n* = 5). qPCR values in **a**, **b**, and **e** were normalized to 18S rRNA. Data in **c** and **d** were assessed by normalized luciferase assays. All data are normalized to BSA control set to 1.0 and plotted as mean ± SEM. Significance was evaluated by **a** two-tailed unpaired Student’s *t* test, **b**–**e** one-way ANOVA with Dunnett’s (**b**, **d**, **e**), or **c** Tukey’s multiple comparisons test. *p ≤ 0.05; **p ≤ 0.01; ***p ≤ 0.001

We therefore continued to search for other regulators of SPARC. To this end, we incubated C2C12 myotubes with several hormonally relevant signaling molecules for skeletal muscle, namely IGF-I, Wnt-3a, tumor necrosis factor (TNF-α), growth/differentiation factor 8 (GDF-8), interferon-gamma (IFN-γ), and the glucocorticoid dexamethasone, and successfully validated the pharmacological treatments using reference genes (Fig. 2a). Importantly, only IGF-I was able to significantly induce SPARC expression (1.8-fold) (Fig. 2b). To test whether IGF-I could regulate SPARC directly, we cloned 1011 basepairs of the human SPARC promoter into a luciferase reporter vector and transfected C2C12 myoblasts with the construct. SPARC promoter activation by IGF-I was significantly induced in a dose-dependent manner (Fig. 2c). We then used chemical inhibitors to the numerous intracellular pathways downstream of IGF-I and silenced RAS-MAPK signaling (PD184352, targeting MEK1/2; NSC23766, targeting Rac GTPase), PI3K-PKB/AKT signaling (LY294002, targeting PI3Kα/δ/β), PLCγ signaling (U73122), STAT signaling (Nifuroxazide), and JAK signaling (VX-509) in the presence of IGF-I. Notably, suppression of IGF-I-activated promoter activity was mostly dependent on the inhibition of the phosphatidylinositol 4,5-bisphospate 3-kinase (PI3K)/protein kinase B (PKB/AKT) pathway using LY294002 (Fig. 2d). The stimulatory effect of IGF-I on SPARC at the mRNA level was also most profoundly reversed by the PI3K inhibitor (Fig. 2e). The inhibitory effect of LY294002 on IGF-I-dependent activation of AKT was confirmed using western blotting (Suppl. Fig. S1). Importantly, inhibition of AKT2 using esiRNA also inhibited the activation of the SPARC promoter (Suppl. Fig. S2a, b). Together, these results indicate that IGF-I/PI3K/AKT2 signaling is an effective and direct inducer of SPARC expression in muscle cells.

Previously, we have shown that IGF-I downregulates miR-29a expression in human myotubes [30] and that miR-29a decreases SPARC expression via binding to its 3′UTR [19]. We therefore asked whether the induction of SPARC by IGF-I is mediated indirectly by downregulation of miR-29a. To this end, we incubated muscle cells with IGF-I in the presence of either a control antagomir or antagomir-29a. As expected, antagomir-29a relieved the suppression of SPARC by miR-29a and upregulated SPARC mRNA levels as compared to control (Suppl. Fig. S3). Importantly, IGF-I was able to increase SPARC expression independent of the presence of the miR-29a inhibitor. These results are in line with our conclusion that IGF-I induces SPARC expression mainly through activation of the SPARC promoter.

**Fig. 3:**
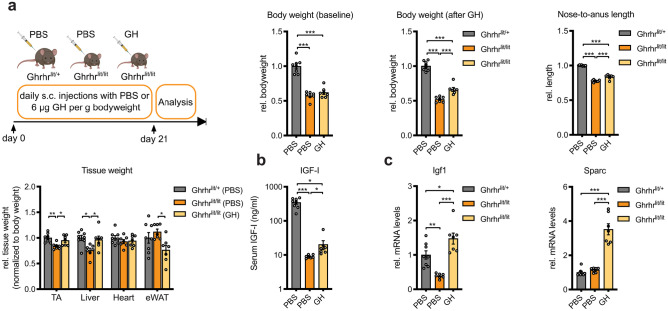
Growth hormone replacement therapy increases SPARC expression in skeletal muscle from Ghrhr-deficient mice. **a** Mice heterozygous or homozygous for the *little* spontaneous mutation in the growth hormone releasing hormone receptor (Ghrhr) gene were injected s c. for 3 weeks once daily with PBS or 6 µg/g bodyweight growth hormone (GH), respectively (*n* = 8 vs. 7 vs. 7). Mice were characterized for body weight and length. After sacrificing, tibialis anterior muscle (TA), liver, heart, and epididymal white adipose tissue (eWAT) were excised and tissue weight was determined. **b** Serum IGF-I concentration as assessed by ELISA. **c** Relative gene expression for IGF-I and SPARC was analyzed in TA muscle using qPCR and normalized to 18S rRNA. All data are plotted as mean ± SEM. Significance was evaluated by **a**, **c** one-way ANOVA with Tukey’s multiple comparisons test and **b** Kruskal–Wallis test. *p ≤ 0.05; **p ≤ 0.01; ***p ≤ 0.001

To test whether IGF-I is required for SPARC regulation in vivo, we employed an animal model for GH deficiency, mice that are deficient for the growth hormone releasing hormone receptor (Ghrhr^lit/lit^). Ghrhr^lit/lit^ mice are homozygous for a missense mutation in the Ghrhr, resulting in less than 5% of normal levels of GH in pituitary and serum and a decrease of 15–20% in serum IGF-I [31]. Ghrhr^lit/+^ heterozygote littermates have normal GH and IGF-I levels [31]. Our study consisted of two PBS-injected control groups, heterozygous and homozygous Ghrhr^lit^ mice, and one group of Ghrhr^lit/lit^ mice receiving GH replacement therapy (GHRT) daily over a period of 3 weeks according to our previously published protocol [30] (Fig. 3a). As expected, Ghrhr^lit/lit^ mice had decreased body weight and length, had lower tissue weight in TA and liver (Fig. 3a), and decreased serum IGF-I and skeletal muscle IGF-I mRNA levels compared to heterozygous Ghrhr^lit/+^ mice (Fig. 3b, c). Three weeks of GHRT in Ghrhr^lit/lit^ mice significantly increased body weight, body length, and tissue weight of TA and liver, whereas epididymal white adipose tissue (eWAT) was significantly reduced compared to control-injected Ghrhr^lit/lit^ mice (Fig. 3a). Serum IGF-I and skeletal muscle IGF-I mRNA levels were both upregulated after GHRT (Fig. 3b, c). Although SPARC expression in skeletal muscle was not different between the Ghrhr^lit/lit^ mice and controls, GHRT drastically induced SPARC mRNA by 3.5-fold compared to control-injected homozygous and heterozygous mice (Fig. 3c). Together, we conclude that reduced IGF-I levels are sufficient to maintain SPARC levels in skeletal muscle and that the activation of the GH/IGF-I axis can robustly stimulate SPARC expression in vivo. To test whether GH directly activates SPARC expression, we incubated GH on muscle cells. However, GH only moderately increased IGF-I expression in C2C12 myotubes and was not sufficient to alter SPARC expression (Suppl. Fig. S4) indicating that the effects that we observed in vivo are likely the result of IGF-I and not GH. To test whether the regulation of SPARC in Ghrhr^lit/lit^ mice depends on FGF-2 signaling as observed in aged muscle [19], we analyzed the expression of FGF-2, the transcription factor Fosl1 which is activated by FGF-2 and miR-29a in these mice. We confirmed that miR-29a levels are downregulated in Ghrhr^lit/lit^ mice by GH treatment (Suppl. Fig. S5) as we have previously reported [30]; however, no significant regulations were observed for FGF-2 and Fosl1 after GH supporting our conclusion that in skeletal muscle, IGF-I and FGF-2 are likely independent regulators of SPARC expression.

**Fig. 4:**
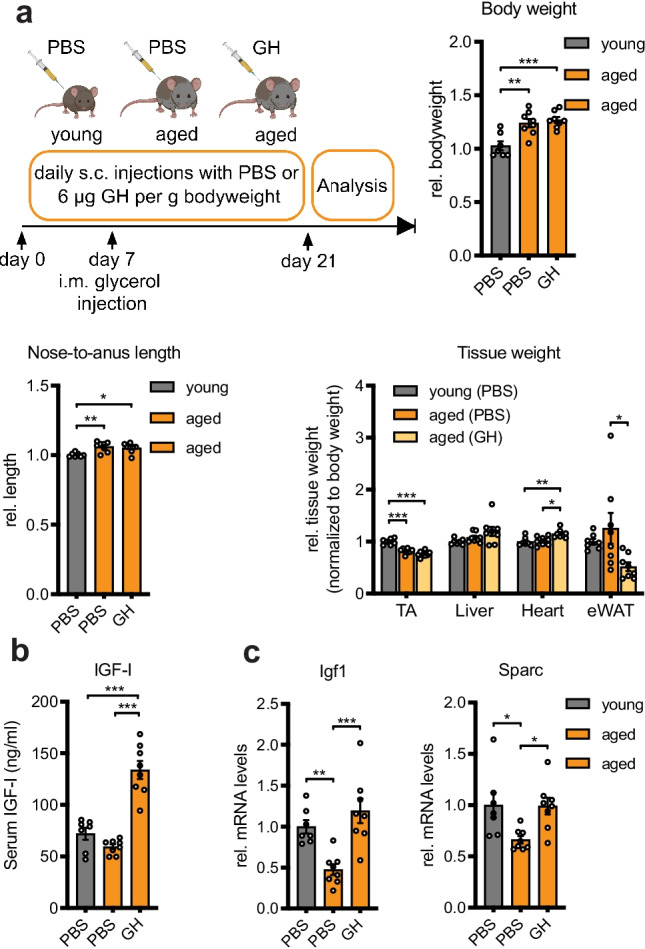
Growth hormone replacement therapy increases IGF-I and restores SPARC levels in aged mice. **a** 2-month-old (young) or 25-month-old (aged) wildtype C57BL/6JRj mice were injected s c. for 3 weeks once daily with PBS and 6 µg/g bodyweight growth hormone (GH), respectively (*n* = 7 vs. 8 vs. 8). On day 7, mice received one glycerol injection in the left tibialis anterior muscle. Mice were characterized for body weight and length. After sacrificing, uninjected tibialis anterior muscle (TA), liver, heart, and epididymal white adipose tissue (eWAT) were excised and tissue weight was determined. **b** Serum IGF-I concentration as assessed by ELISA. **c** Relative gene expression for IGF-I and SPARC was analyzed in uninjected TA muscle using qPCR and normalized to 18S rRNA. All data are plotted as mean ± SEM. Significance was evaluated by one-way ANOVA with Tukey’s multiple comparisons test. *p ≤ 0.05; **p ≤ 0.01; ***p ≤ 0.001

With increasing age, GH secretion declines resulting in a decrease in circulating IGF-I in both human and rodent models. We have previously shown that SPARC expression is markedly decreased in aged skeletal muscle from both mice and humans [19]. We therefore asked whether the induction of IGF-I using GH therapy in aged mice could restore the decreased SPARC levels. To this end, we treated 25-month-old (aged) mice with GH and used PBS-injected 2-month-old (young) and PBS-injected (aged) mice as control (Fig. 4a). As expected, aged mice had increased body weight and body length, while TA weight was significantly reduced compared to young mice (Fig. 4a). GH treatment did not affect TA weight, but increased the relative tissue weight of the heart and decreased the weight of a classical fat depot, the epididymal white adipose tissue (eWAT) (Fig. 4a). Serum IGF-I levels were markedly induced by GH treatment (Fig. 4b). IGF-I and SPARC expression were decreased in the skeletal muscle of aged mice compared to controls, as we have previously shown [19] (Fig. 4c). Importantly, GH therapy increased both IGF-I and SPARC expression to similar levels as observed in young mice (Fig. 4c). These results demonstrate that GH/IGF-I signaling is able to restore SPARC levels in aged skeletal muscle in vivo.

**Fig. 5:**
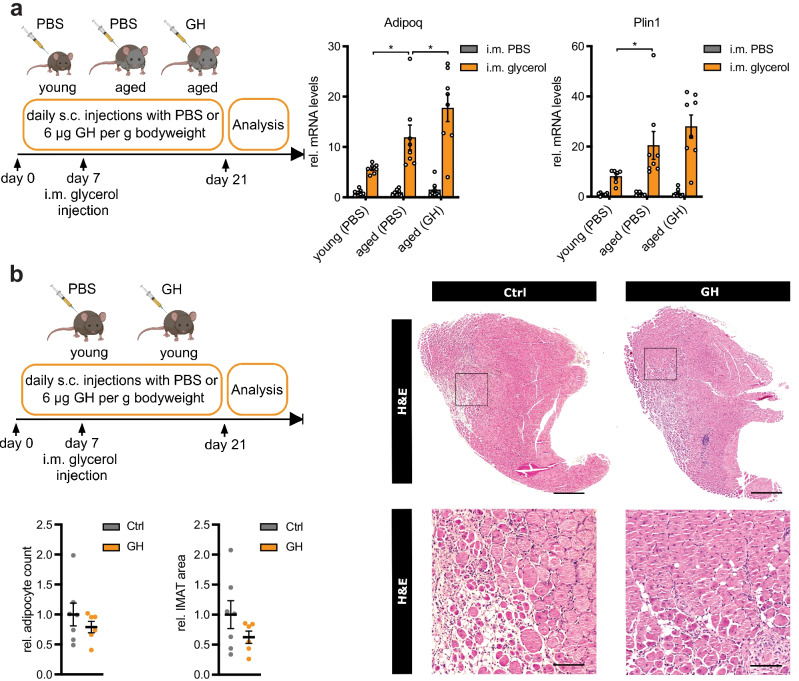
GH therapy does not prevent IMAT formation in the glycerol model in aged or young mice. **a** 2-month-old (young) or 25-month-old (aged) wildtype C57BL/6JRj mice were injected s c. for 3 weeks once daily with PBS and 6 µg/g bodyweight growth hormone (GH), respectively (*n* = 7 vs. 8 vs. 8). On day 7, mice received one glycerol injection in the left tibialis anterior muscle. Relative gene expression for Adipoq and Plin1 was analyzed using qPCR in uninjected and glycerol-injected TA muscles and normalized to 18S rRNA. **b** 2-month-old (young) wildtype C57BL/6JRj mice were injected s c. for 3 weeks once daily with PBS (Ctrl) and 6 µg/g bodyweight growth hormone (GH), respectively (*n* = 7 vs. 6). On day 7, mice received one glycerol injection in the left tibialis anterior muscle. Relative adipocyte count and IMAT were analyzed on H&E stained paraffin sections from glycerol-injected TA muscles. Black squares indicate areas of magnification (bottom panel). Scale bar: 0.5 mm, top panel; 100 µm, bottom panel. All data are plotted as mean ± SEM. Significance was evaluated by **a** one-way ANOVA with Tukey’s multiple comparisons test or **b** two-tailed unpaired Student’s *t* test

We have previously shown that overexpression of SPARC using adeno-associated virus in skeletal muscle from mice decreases IMAT formation using the glycerol model of muscle regeneration and that a reduction of SPARC expression in skeletal muscle from aged humans by ~ 50% was associated with increased IMAT [19]. Consequently, we asked whether restoring SPARC levels in aged mice using GH would be sufficient to prevent intramuscular adipogenesis. To this end, we compared the expression of adipocyte markers in the tibialis anterior muscle of aged mice with or without GH therapy and young control mice (Fig. 5a). Glycerol injections markedly increased the expression of adiponectin (Adipoq) and perilipin (Plin1) (Fig. 5a). Aged mice reached significantly higher levels of both adipocyte markers, but GH therapy was not able to reduce this induction (Fig. 5a). The effect of GH treatment on muscle histology was tested in young mice. GH therapy did also not significantly alter the relative adipocyte count or IMAT area (Fig. 5b).

**Fig. 6:**
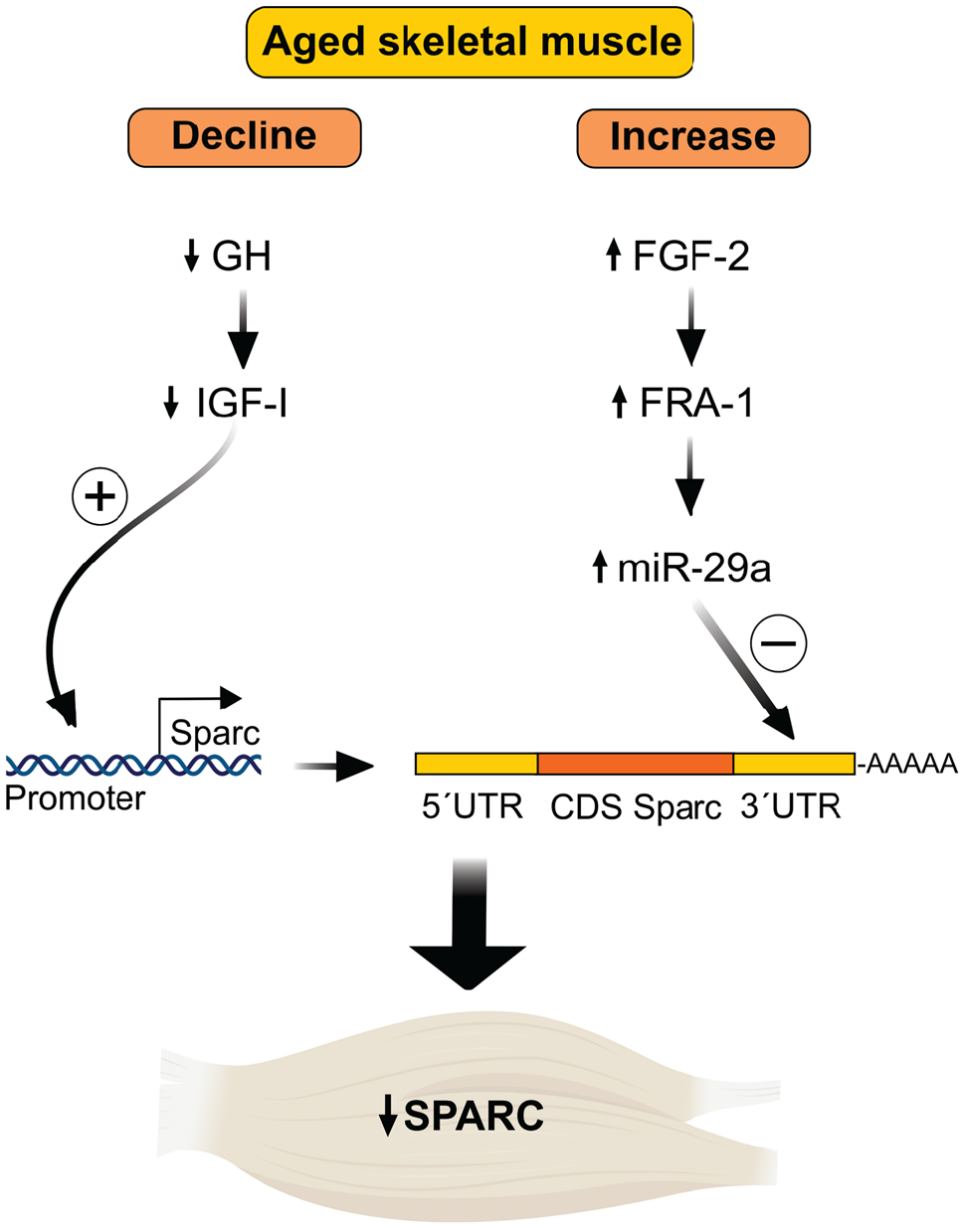
Hypothetical model for growth factor signaling regulating SPARC in aged skeletal muscle. Growth hormone (GH)/insulin-like growth factor I (IGF-I) signaling is decreased during aging, while fibroblast growth factor-2 (FGF-2) signaling is activated. The GH/IGF-I axis induces SPARC by direct activation of the SPARC promoter, while FGF-2 decreases SPARC levels indirectly by increased binding of miR-29a to the 3′UTR of SPARC. By that, both growth factor signaling pathways contribute to decreased SPARC expression in aged skeletal muscle

Together, we conclude that GH/IGF-I signaling is able to restore SPARC levels in skeletal muscle from aged mice. We suggest that both GH/IGF-I and FGF-2 regulate SPARC in skeletal muscle during aging (Fig. 6). Induction of SPARC expression using GH treatment was not sufficient to reverse the increased IMAT in aged skeletal muscle in the glycerol model.

## Discussion

Skeletal muscle can secrete hundreds of factors, such as peptides, growth factors, and cytokines that affect different aspects of inflammation, regeneration, and metabolism [2]. The myokine SPARC is a novel regulator of IMAT in aged skeletal muscle [19] and therefore an important therapeutic target. Here, we demonstrate that IGF-I signaling regulates SPARC expression in muscle cells in vitro and in vivo and that activation of the GH/IGF-I axis is able to restore the reduced expression of SPARC in aged skeletal muscle. Although GH therapy in young and aged mice failed to prevent IMAT in the glycerol model, inducing SPARC using exercise or activation of IGF-I signaling might still hold promises in patients at risk for IMAT formation during aging.

IGF-I is a critical growth factor and primarily regulates muscle mass through activation of protein synthesis on the one hand [32] and inhibition of protein breakdown pathways on the other [33, 34]. Here, we provide for the first time evidence that IGF-I also participates in the regulation of the myokine SPARC in skeletal muscle. Our results demonstrate a direct link between IGF-I and the expression of SPARC in skeletal muscle. IGF-I activated in a dose-dependent manner the SPARC promoter and consequently increased the SPARC transcript in muscle cells in vitro.

SPARC is an important inhibitor of adipocyte differentiation. In skeletal muscle, SPARC inhibits the formation of adipocytes by preventing the differentiation of fibro/adipogenic progenitors (FAPs) [19]. We have shown that FGF-2-dependent signaling negatively regulates SPARC expression. Here, we were especially interested in pathways that activate SPARC expression and that could provide alternative therapeutic avenues to prevent IMAT formation during aging. Outside skeletal muscle, SPARC expression is regulated by several hormonal cues and metabolites in cell lines, visceral adipose tissue explants, and the heart, and we have previously shown that GHRT is associated with increased SPARC levels in skeletal muscle of GH-deficient mice [30]. In the current study, we extended these findings to include a screen of six different signaling pathways in muscle cells combined with promoter assays using the human SPARC promoter. Through stimulation of muscle cells with recombinant proteins and a glucocorticoid, we identified IGF-I as a main effector for SPARC expression and showed that IGF-I-mediated activation of SPARC promoter activity was mostly dependent on the inhibition of the PI3K signaling pathway. That IGF-I regulates gene expression through PI3K signaling is plausible since PKB/AKT is an important downstream effector of PI3K and the phenotypes of PKB/AKT knockout mice are strikingly similar to the phenotypes of IGF-I receptor-deficient mice [35]. These results together with our data indicate that PI3K/PKB/AKT signaling serves as the most critical downstream effector of the IGF-I receptor. Interestingly, the transcription factor c-Jun was recently found to be a key transcriptional target of the PI3K/PKB/AKT/mTORC1 signaling axis in muscle satellite cells [36] and c-Jun overexpression in MCF7 breast cancer cells increased SPARC levels [37]. However, the identification of the transcription factor(s) mediating SPARC expression downstream of IGF-I in skeletal muscle needs further investigation.

A major strength of our study is that we employed two different in vivo models for deficiency of the GH/IGF-I axis. From the mouse model for GH deficiency (Ghrhr^lit/lit^), we ascertained that although reduced IGF-I levels are sufficient to maintain SPARC expression in skeletal muscle, SPARC is under the control of GH since systemic GH treatment robustly induces SPARC and IGF-I in the tibialis muscle. There is evidence that the effects of the GH/IGF-I axis on skeletal muscle are predominantly due to IGF-I signaling since GH administration led to increased muscle weight and myofiber cross-sectional area in wildtype mice, but not in mice expressing a dominant-negative IGF-I receptor in muscle [38]. In addition, we show that GH does not regulate SPARC expression in muscle cells in vitro. Therefore, GHRT in Ghrhr-deficient mice is an appropriate model to study the effects of IGF-I on skeletal muscle and the increase of SPARC after GHRT in Ghrhr^lit/lit^ mice is likely caused by the action of IGF-I. The second mouse model we used in our study is aged mice. The age-dependent, exponential decline in GH has been considered to be primarily due to reduced hypothalamic secretion of GHRH, resulting in decreased circulating IGF-I levels [39]. In humans, GH synthesis declines between 14% per decade [40] and 50% every 7 years [41]. In our aged mouse model, we detected a similar decrease in IGF-I mRNA levels as we and others observed in the Ghrhr^lit/lit^ mice [42]. However, SPARC levels were reduced in aged skeletal muscle but not in muscle from the Ghrhr-deficient mice. These results suggest that reduced IGF-I signaling is not the primary cause for the decreased SPARC levels in aged muscle and support the importance of the FGF-2-dependent inhibition of SPARC that we have previously described [19]. We propose that IGF-I and FGF-2 signaling converge on SPARC regulation in skeletal muscle as outlined in Fig. 6.

In our study, we observed only a mild increase in circulating SPARC levels immediately after an acute exercise bout (90 min at 23 cm s^−1^) in mice. At the mRNA level, we observed a significant regulation of SPARC expression only in the gastrocnemius muscle. Higher gene regulation in gastrocnemius muscle compared to tibialis anterior is in line with the plantarflexor muscle group being more affected by exercise intervention in mice than the tibialis anterior muscle [43]. Indeed, a previous study reported that a single bout of exercise can increase SPARC protein approximately twofold in the gastrocnemius muscle of mice [21]. The same study reported an increase of SPARC mRNA in the gastrocnemius muscle by approximately 2.5-fold after a 4-week exercise protocol (5 times per week) without changes in steady state SPARC levels in plasma [21]. In humans, several studies reported increased SPARC mRNA levels in skeletal muscle after 6- to 12-week exercise training programs [22, 23, 44–46], while two of these studies did not detect different intramuscular SPARC mRNA levels after acute exercise [23, 45]. Together, we conclude that chronic exercise might be more efficient to increase skeletal muscle SPARC levels compared to acute exercise in mice and humans.

GH administration is lipolytic and causes an increase in serum-free fatty acids partly due to increased hormone-sensitive lipase activity. Accordingly, we have observed a decrease in the eWAT mass in both our GH deficiency models. In old rats, GH therapy restored IGF-I values to the level of young rats and improved sarcopenia [47]. GH administration in healthy 60- to 85-year-old men reduced fat mass, increased lean body mass, and elevated bone mineral density [48, 49]. Using GH, we could successfully restore decreased SPARC levels in aged mice; thus, our results add to the potential pharmacological benefits of a GH therapy in carefully selected patients with GH deficiency. Whether GH therapy would also prevent IMAT formation in human skeletal muscle remains to be elucidated.

Using the non-physiological glycerol model to assess the effect of GH therapy on IMAT is a limitation of our study. However, the glycerol model is the most frequently used model for IMAT in mice since intramuscular adipocyte formation in mice is rare and usually not observed in other models such as denervation atrophy or cancer cachexia. The amount of adipocytes observed in the glycerol model depends on the mouse background which might also influence the effect of SPARC on IMAT formation. In the present study, the aged mice and their young controls were provided in the BL/6JRj background which yielded less IMAT formation compared to what we have previously observed in mice with the Sv129 background (unpublished data) (Fig. 5B). Inducing SPARC using GH in the Sv129 mouse background might yield different results. It is also possible that we did not reach a threshold for SPARC that is required for therapeutic effects. In our previous study, we used a 5.5-fold induction of SPARC expression in skeletal muscle to decrease IMAT formation in the glycerol model [19]. Finally, the fact that inducing SPARC using GH did not mirror the decreased IMAT formation that we observed after AAV gene delivery of SPARC into the tibialis anterior muscle [19] could be related to the effects downstream of GH that act antagonistic to SPARC. We therefore hypothesize that increasing SPARC using tailored exercise protocols or directly via IGF-I could still provide important benefits on reducing IMAT in humans during aging. GH therapy in skeletal muscle-specific SPARC KO models might help to discriminate opposing effects of GH and SPARC on IMAT.

Together, we provide evidence for the regulation of the myokine SPARC in aged skeletal muscle by GH/IGF-I. Targeting SPARC using exercise or activation of IGF-I signaling may be a promising therapy for individuals who are at particular risk for the formation of IMAT.

## Supplementary Information

Below is the link to the electronic supplementary material.

Supplementary file1 (PDF 418 KB)

## Data Availability

The authors declare that all data supporting the findings of this study are available within the article or from the corresponding author upon reasonable request.
