# Supplementary material for: Growth hormone/IGF-I-dependent signaling restores decreased expression of the myokine SPARC in aged skeletal muscle
Source: J Mol Med (Berl). 2022 Sep 30;100(11):1647–58. doi: 10.1007/s00109-022-02260-w (PMC9592655; doi:10.1007/s00109-022-02260-w)
Supplement: Supplementary file 1 — Supplementary file1 (PDF 418 KB) [file 109_2022_2260_MOESM1_ESM.pdf]

Supplementary Information

Growth hormone/IGF-I-dependent signaling restores decreased expression of the myokine Sparc in aged skeletal muscle

Sebastian Mathes[1,2], Alexandra Fahrner[1,2], Edlira Luca[1] and Jan Krützfeldt[1,2].

[1]Department of Endocrinology, Diabetology, and Clinical Nutrition, University Hospital Zurich (USZ) and University of Zurich (UZH), 8091 Zurich, Switzerland,

[2]Life Science Zurich Graduate School, Biomedicine, University of Zurich, 8057 Zurich, Switzerland

Corresponding author and person to whom reprint requests should be addressed to:

Jan Krützfeldt, MD, University Hospital Zurich, Department of Endocrinology, Diabetes, and Clinical Nutrition, Rämistrasse 100, 8091 Zurich, Switzerland. Phone: +41 (0)44 255 36 27, Fax: +41 (0)44 255 9741, E-mail: jan.kruetzfeldt@usz.ch

Supplemental Figures

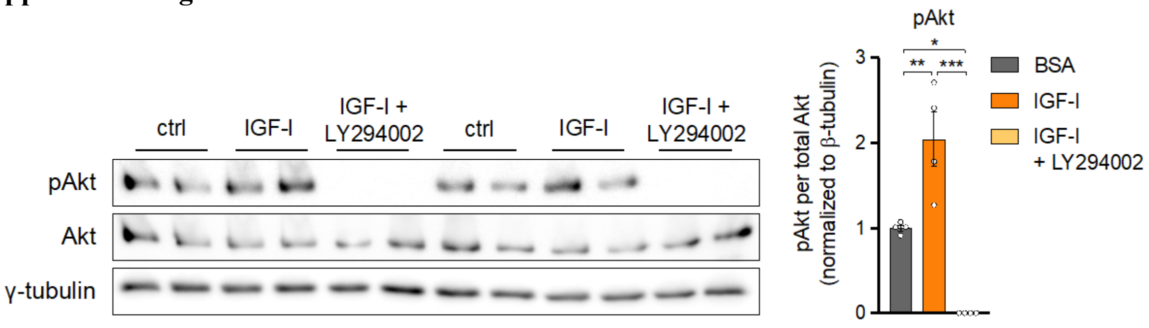

Fig. S1. LY294002 inhibits IGF-I-dependent activation of AKT. Immunoblot depicting inhibition of IGF-I-dependent activation of AKT by LY294002. Band densities were quantified relative to total AKT protein normalized to γ-tubulin (n = 4). All data are plotted as mean ± SEM. Significance was evaluated by one-way ANOVA with Tukey´s multiple comparisons test. *$p \leq 0.05$; **$p \leq 0.01$; ***$p \leq 0.001$.

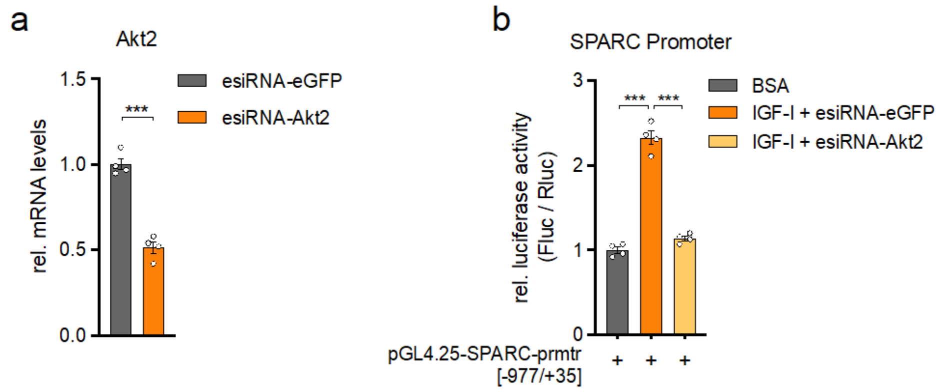

Fig. S2. Knockdown of Akt2 inhibits the activation of the SPARC promoter. (**a**) Akt2 expression normalized to 18S rRNA in C2C12 myoblasts after knockdown of Akt2 (n = 4). SPARC promoter activity after treatment with IGF-I and/or knockdown of Akt2 (n = 4). All data are plotted as mean ± SEM. Significance was evaluated by (**a**) two-tailed unpaired Student`s t-test, or (**b**) one-way ANOVA with Tukey´s multiple comparisons test. ***$p \leq 0.001$.

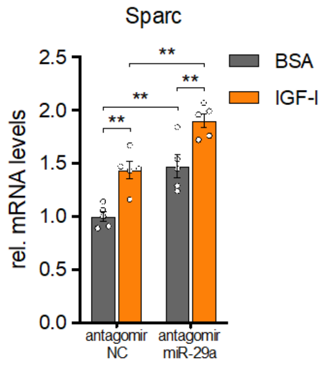

Fig. S3. IGF-I regulates Sparc independent of miR-29a. Sparc expression in C2C12 myotubes after treatment with IGF-I and inhibition of miR-29a using antagomirs. qPCR values were normalized to 18S rRNA and plotted relative to antagomir negative control (NC; n = 5). All data are plotted as mean ± SEM. Significance was evaluated by one-way ANOVA with Tukey´s multiple comparisons test. **$p \leq 0.01$.

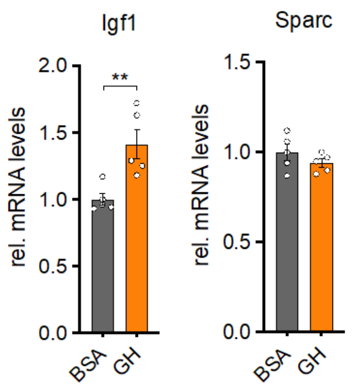

Fig. S4. GH induces Igf1 but not Sparc expression. Igf1 and Sparc expression in C2C12 myotubes after treatment with GH for 48 h (n = 5). qPCR values were normalized to 18S rRNA. All data are plotted as mean ± SEM. Significance was evaluated by two-tailed unpaired Student`s t-test. $^{**}p \leq 0.01$.

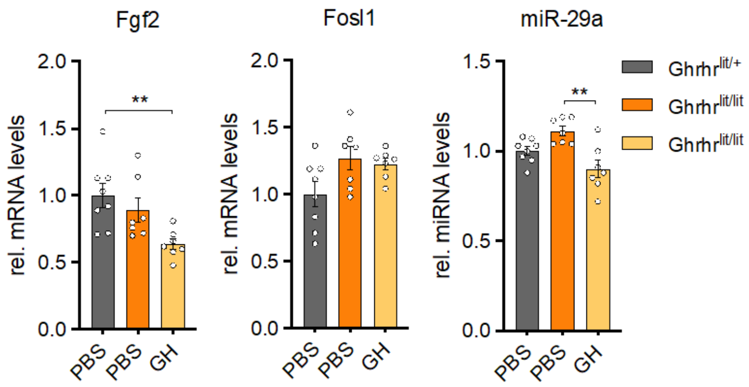

Fig. S5. Growth hormone replacement therapy decreases miR-29a but does not regulate Fgf2 and Fosl1. Fgf2, Fosl1 and miR-29a expression in TA muscle of mice heterozygous or homozygous for the *little* spontaneous mutation in the growth hormone releasing hormone receptor (Ghrhr) gene. Mice were injected s c. for 3 weeks once daily with PBS or 6 µg/g bodyweight growth hormone (GH), respectively (n = 8 *vs*. 7 *vs*. 7). qPCR values were normalized to 18S rRNA (Fgf2, Fosl1) or snoRNA234. All data are plotted as mean ± SEM. Significance was evaluated by one-way ANOVA with Tukey´s multiple comparisons test. $^{**}p \leq 0.01$.

Supplemental Table

Table S1. List of oligonucleotides used in the present study.

**Name**	**Sequence (5´→ 3´)**	**Application**
h_SPARC-promoter [-977/+35]	FWD: TTTTTGAGGACAAGGACCAGGTAT<br>REV: GCTCTCCGGGCAGTCTGAAG	Cloning
m_18S rRNA	FWD: GACACGGACAGGATTGACAGATTG<br>REV: AAATCGCTCCACCAACTAAGAACG	qPCR
m_Sparc	FWD: TTCAGACCGCCAGAACTCTT<br>REV: CCAGGCAAAGGAGAAAGAAG	qPCR
m_Igfbp3	FWD: TGCCGCAGAGAAATGGAGGACA<br>REV: AGGGCGGCACTGCTTCTTCTTA	qPCR
m_Ppargc1a	FWD: GCGAACCTTAAGTGTGGAAC<br>REV: CACCACGGTCTTGCAAGAGG	qPCR
m_Col3a1	FWD: GGAACCTGGTTTCTTCTCACC<br>REV: TAGGACTGACCAAGGTGGCT	qPCR
m_Axin2	FWD: ACTGACCGACGATTCCATGT<br>REV: TGCATCTCTCTCTGGAGCTG	qPCR
m_Fbxo32	FWD: AAGGAGCGCCATGGATACT<br>REV: TCAGGGATGTGAGCTGTGAC	qPCR
m_Nfkb1	FWD: AGAGGGGATTTCGATTCCGC<br>REV: CCTGTGGGTAGGATTTCTTGTTC	qPCR
m_Igf1	FWD: TTACTTCAACAAGCCCACAGG<br>REV: GCAACACTCATCCACAATGC	qPCR
m_Adipoq	FWD: TGTTCCTCTTAATCCTGCCCA<br>REV: CCAACCTGCACAAGTTCCCTT	qPCR
m_Plin1	FWD: CAAGCACCTCTGACAAGGTTC<br>REV: GTTGGCGGCATATTCTGCTG	qPCR
m_Igf1	FWD: TTACTTCAACAAGCCCACAGG<br>REV: GCAACACTCATCCACAATGC	qPCR
m_Fgf2	FWD: AGAAGAGCGACCCACACG<br>REV: GGCACACACTCCCTTGATAGA	qPCR
m_Fosl1	FWD: ATGTACCGAGACTACGGGGAA<br>REV: CTGCTGCTGTCGATGCTTG	qPCR
m_Akt2	FWD: ACGTGGTGAATACATCAAGACC<br>REV: GGGCCTCTCCTTATACCCAAT	qPCR

Supplemental Material and Methods

Protein extraction and western blot. C2C12 myoblasts were lysed in RIPA Buffer (25 mM Tris HCl pH7.6, 150 mM NaCl, 1% NP-40, 1% sodium deoxycholate, 0.1% SDS) supplemented with protease (Complete, Roche) and phosphatase (PhosSTOP, Roche) inhibitor cocktails. Lysates were cleared by centrifugation at 14,000 × g for 20 min at 4 °C. Protein concentrations were determined using Pierce BCA Protein Assay Kit (Thermo Sientific). Equal amounts of protein (25 µg) were separated by SDS-PAGE, transferred onto Protran Nitrocellulose Membranes (GE Healthcare) using eBlot L1 Fast Wet Transfer System (GenScript), followed by incubation with 1:1000 primary antibodies (pAKT, #4058, AKT, #2920 both Cell Signaling; γ-tubulin #T-5326, Sigma) according to manufacturer´s instruction. Signals of respective IgG horseradish peroxidase-conjugated secondary antibodies were visualized on a LAS-3000 Luminescent Image Analyzer (Fujifilm) using Lumi-Light Western Blotting Substrate (Roche). Specific protein bands were densitometrically quantified using ImageJ (v 1.52q). Quantitative data were obtained by normalizing total AKT values for γ-tubulin. pAKT values were subsequently calculated relative to normalized AKT values. Densitometry was performed from 4 independent samples. LY294002 is a widely used inhibitor of PI3K that inactivates AKT/PKB. The verification experiment of the pharmacological inhibition of PKB/AKT by LY294002 was therefore only performed once.

TaqMan RT-qPCT. 10 ng of total RNA was reverse-transcribed using the TaqMan MicroRNA Reverse Transcription Kit (Applied Biosystems). Quantitative RT-PCR for miRNA was performed on a 7500 FAST Real-time PCR system (Applied Biosystems) using TaqMan Fast Universal PCR Master Mix, no AmpErase UNG (Applied Biosystems). The levels of miRNA were calculated using the ΔΔCt method and snoRNA234 for normalization. TaqMan assays for miR-29a (TM: 002112) and snoRNA234 (TM: 001234) were purchased from Applied Biosystems.

Full unedited blots

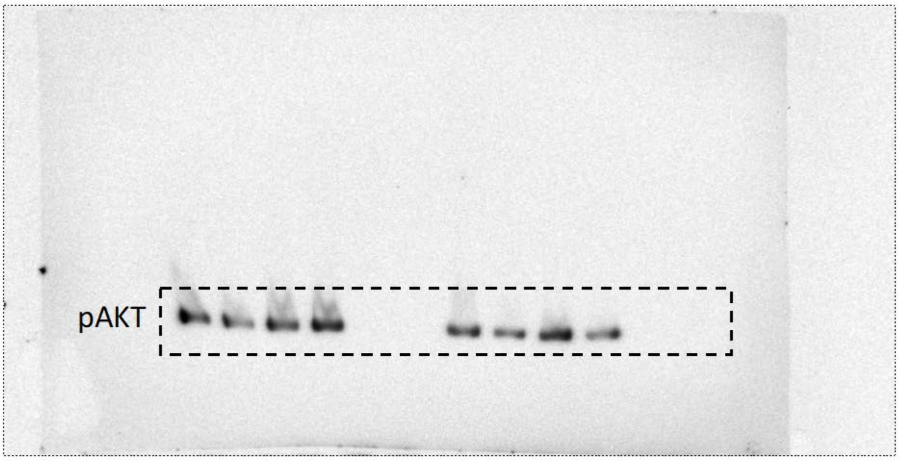

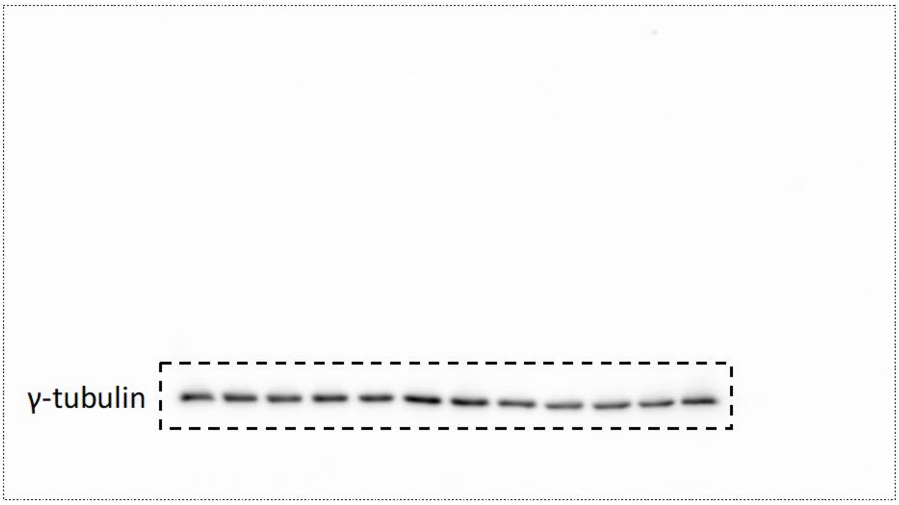

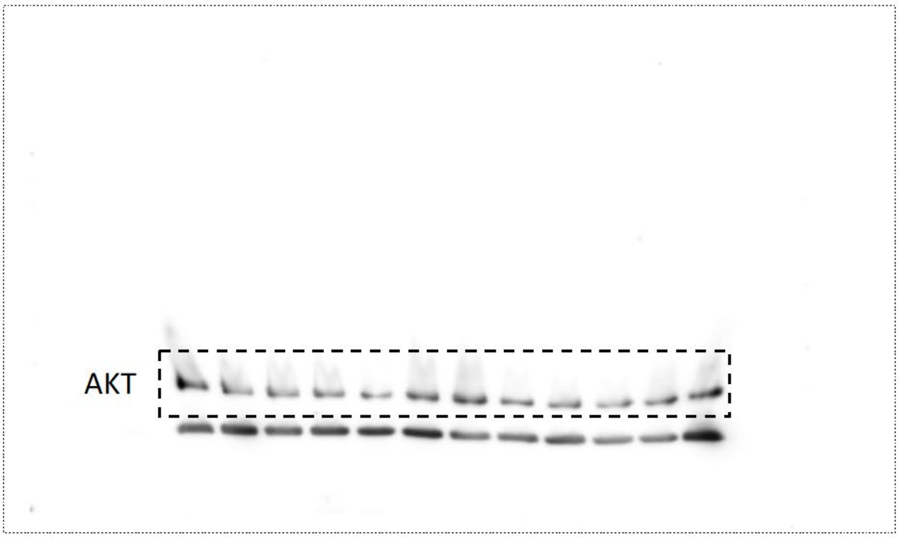

Full unedited membranes for Fig. S1. The membrane was first incubated with anti-pAKT (development time 30 min). The same membrane was then incubated with anti-γ-tubulin (development time 30s). The same membrane was further incubated with anti-AKT (development time 5 min). The images show the full uncropped/unedited images of the original blot.
